# Electron Doping Effect in the Resistive Switching Properties of Al/Gd_1–*x*_Ca_*x*_MnO_3_/Au Memristor Devices

**DOI:** 10.1021/acsami.1c02963

**Published:** 2021-04-09

**Authors:** Ville Lähteenlahti, Alejandro Schulman, Azar Beiranvand, Hannu Huhtinen, Petriina Paturi

**Affiliations:** Wihuri Physical Laboratory, Department of Physics and Astronomy, University of Turku, FI-20014 Turku, Finland

**Keywords:** Resistive switching, Perovskite oxides, Poole-Frenkel, Gd_(1−*x*)_Ca_*x*_MnO_3_ and Memristor

## Abstract

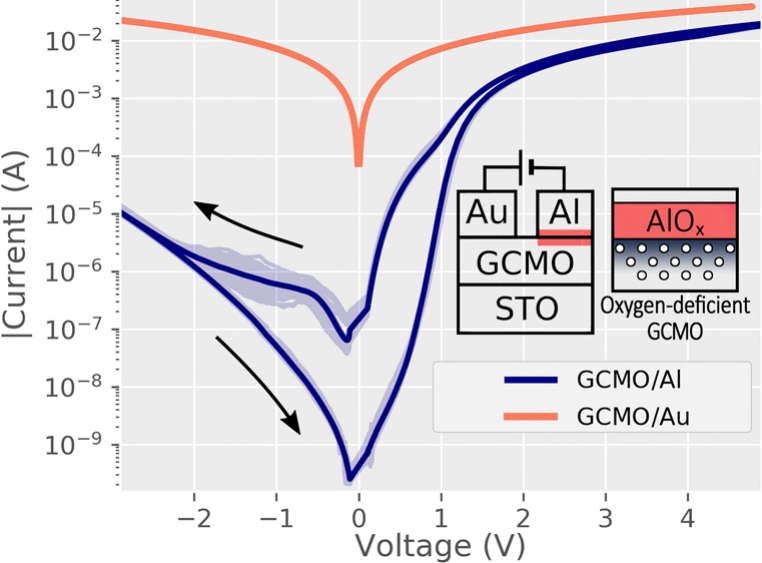

We report on the resistive switching (RS) properties of Al/Gd_1–*x*_Ca_*x*_MnO_3_ (GCMO)/Au thin-film memristors. The devices were studied over the whole calcium substitution range *x* as a function of electrical field and temperature. The RS properties were found to be highly dependent on the Ca substitution. The optimal concentration was determined to be near *x* = 0.9, which is higher than the values reported for other similar manganite-based devices. We utilize an equivalent circuit model which accounts for the obtained results and allows us to determine that the electrical conduction properties of the devices are dominated by the Poole–Frenkel conduction mechanism for all compositions. The model also shows that lower trap energy values are associated with better RS properties. Our results indicate that the main RS properties of Al/GCMO/Au devices are comparable to those of other similar manganite-based materials, but there are marked differences in the switching behavior, which encourage further exploration of mixed-valence perovskite manganites for RS applications.

## Introduction

In recent years, many memory technologies have emerged with the goal to replace traditional charge storage-based memory technologies, which are approaching their physical limits of scalability. The most prominent of them are random access memories based on phase change, resistivity change (RRAM), spin-transfer torque magnetoresistance, and ferroelectricity. Out of them, the resistive switching (RS)-based RRAM has been recently gaining traction due to its promising characteristics. The research on RRAM devices has been active since the early 00’s, and many candidate materials and explanations for the phenomenon have been proposed.^1−6^

In RS, the resistance of the device can be controlled with an electric field in a nonvolatile way, enabling two-terminal devices with good spatial scalability and minimal supporting circuitry. The phenomenon is based on controllable and reversible structural modification, which is most commonly achieved by the movement of oxygen vacancies.^1^ This contrasts with conventional transistor-based memory technologies, where the state is stored nonstructurally in the electrical charge. The advances in RS research have led to device demonstrations with high switching speeds, robust endurance, low power consumption, and compatibility with existing electronics.^7,8^ RS devices have also found use in information processing applications, such as neuromorphic computing,^9^ matrix-vector multiplication,^10^ and convolution kernel operations,^11,12^ which pave the way for efficient hardware-based machine learning.

A lot of promising research, including many of the mentioned demonstrations have been performed using mixed-valence perovskite manganites R_1–*x*_A_*x*_MnO_3_, where R is a rare-earth cation and A is an alkali or alkaline earth cation. These compounds are versatile for RS applications since as the concentration of divalent A cations *x* changes, the compounds go through significant changes in the RS properties. Manganite-based RS devices have been shown to possess good device-to-device variability,^13^ forming-less operation,^14^ and well-controlled analogue resistance states,^15^ all of which are beneficial in building neuromorphic circuits.^16^

The most commonly studied manganite compound is Pr_1–*x*_Ca_*x*_MnO_3_ (PCMO) with *x* = 0.3, which has been proven suitable for both memory and neuromorphic applications with well-performing single devices and large-scale crossbar arrays integrated into existing semiconductor technology.^8,9,13,14,17−19^ Other members of the mixed-valence manganite family, such as La_1–*x*_Ca_*x*_MnO_3_^20^ and La_1–*x*_Sr_*x*_MnO_3_ (LSMO),^21^ share a similar RS mechanism^22^ but have distinct RS characteristics which vary over the calcium substitution range. Currently, many of the possible cation combinations remain unexplored, and the optimal manganite material for neuromorphic applications remains undecided.

**Figure 1:**
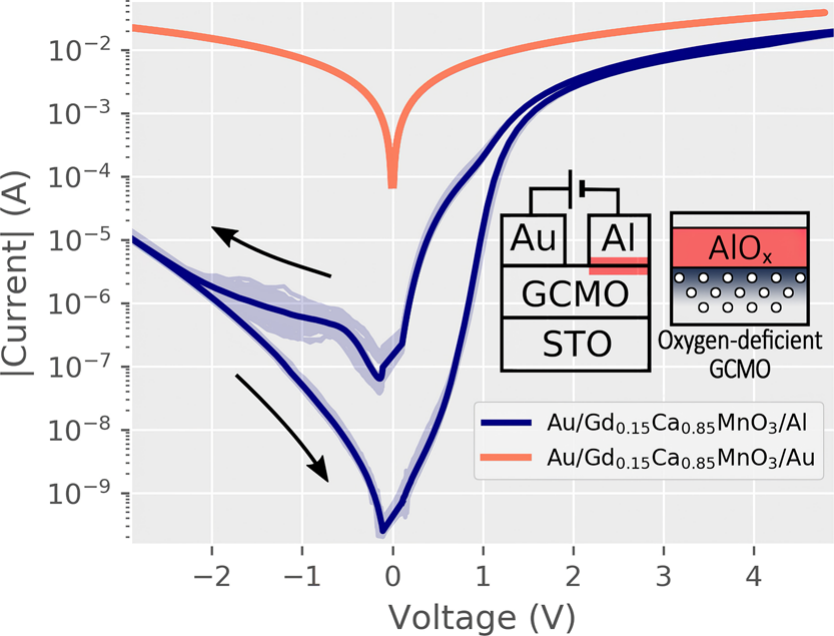
Averaged *I*(*V*) curves for Al/GCMO/Au and Au/GCMO/Au interfaces with *x* = 0.85 over 50 repeated measurements; the lighter shade indicates individual measurements. The conduction in Au interfaces is Ohmic, and the switching happens at the Al interface. The inset shows the measurement configuration and the active aluminum switching interface with the oxygen-deficient GCMO region and the AlO_*x*_ barrier layer.

Manganite-based RS devices usually consist of metal-oxide-metal layer structures, in which one of the metal-oxide interfaces acts as an active electrode where the RS happens. The active interface is made from a reactive material, such as Al, Ti, or TiN, which forms an insulating oxide barrier layer with the manganite^1,23^ (Figure 1 inset). The properties of the barrier are modified with a controlled electric field. The field causes oxygen vacancy drift at the interface and the perovskite structure in the vicinity of the interface.^24^ These changes in turn enable nonvolatile control of the resistivity and capacitance of the RS device.^1,22^

In this article, we report the RS properties of Gd_1–*x*_Ca_*x*_MnO_3_ (GCMO) (0 ≤ *x* ≤ 1) for the first time. GCMO is studied with aluminum as the active electrode, and the resulting devices are characterized and analyzed over the whole calcium substitution range as a function of temperature and electric field. As a mixed-valence perovskite manganite, GCMO is structurally similar to PCMO, but the Gd cation has a smaller ionic radius than Pr, which entails a more distorted structure. This causes a lower bandwidth and more insulating bulk, which is beneficial in RS applications due to lower leakage currents. The calcium concentration series of the GCMO is found to have optimal RS properties at high calcium doping, which cannot be predicted from previous studies on other manganite materials.

## Experimental Details

Gd_1–*x*_Ca_*x*_MnO_3_ thin films with a thickness of approximately 100 nm were deposited on 5 × 5 × 0.5 mm^3^ (100) SrTiO_3_ (Crystal GmbH) substrates by pulsed laser deposition using λ = 308 nm XeCl-laser. The pulse duration was 25 ns, repetition rate 5 Hz, laser fluence 2 J/cm^2^, and the pressure of flowing oxygen in the chamber 0.175 Torr. Each sample was deposited using 1500 pulses. The films were grown at 700 °C with *in situ* postannealing treatment of 10 min in atmospheric oxygen pressure. The films were made from deposition targets which were fabricated for the whole calcium range in increments of 0.1 and for concentrations *x* = 0.85 and *x* = 0.95.^25^ The grown films were verified to be well-crystallized and epitaxially textured by X-ray diffraction (XRD). The elemental compositions of the samples were verified using energy-dispersive spectroscopy, which showed compositions similar to the nominal values and no systematic deviation.^26^ More details on the fabrication process and XRD analysis can be found in refs (27) and (28).

The Al/GCMO/Au RS devices were made by depositing 0.5 mm diameter metal electrodes on top of the films using room-temperature Ar-ion DC sputtering. Wiring was done with a wedge wire bonder using a 40 μm diameter Al wire. The separation distance between the electrodes was approximately 200 μm. The reproducibility of the RS was confirmed by testing multiple thin-film devices made on separate substrates. The characteristics were found to be consistently similar.

Electrical measurements were performed in a planar configuration as schematized in the inset of Figure 1 using a Keithley 2614b sourcemeter. The *I*(*V*) loops were measured by sweeping the voltage in a sequence of steps 0 → *V*_max_ → −*V*_min_ → 0 with logarithmic amplitude progression, 100 ms step width, and a 100 ms low-voltage read between each step. Each room-temperature loop measurement was repeated 50 times in order to confirm the stability of the RS device. The voltage amplitudes for inducing high resistance state (HRS) and low resistance state (LRS) were chosen to give maximum switching ratio without damaging the device. The voltages represent extremes, and the optimal values for applications could be lower. Datapoints were measured during and after the writing pulse. The low-voltage current read was set at 450 mV, which was determined to be in the Ohmic region for all calcium concentrations.

## Results and Discussion

**Figure 2:**
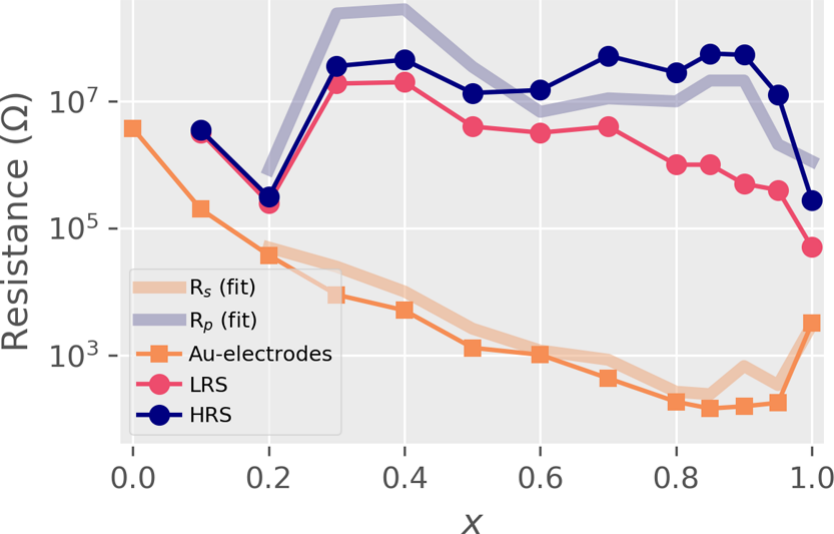
Resistance values for GCMO with Ohmic interfaces (Au electrodes) and Al interfaces (HRS and LRS) over the calcium substitution range *x* at room temperature. *R*_s_ and *R*_p_ show results from the conduction model fit (Figure 6 inset); *R*_s_ coincides with the Ohmic interface, and *R*_p_ coincides with the HRS.

The fabricated devices were designed to have only one active interface. This was achieved by using gold and aluminum electrodes. The gold interface does not contribute to the switching and forms an Ohmic interface with GCMO (Figures 1 and 2). Similarly, when Kelvin probe measurements are done on the bulk of the GCMO thin film, the conduction is linear and the switching is not present, narrowing the switching phenomenon to the vicinity of the aluminum interface.

The aluminum electrode forms a rectifying AlO_*x*_ layer and depletes the perovskite structure of GCMO from oxygen in the region near the interface (Figure 1 inset). The effects of the interface layer and oxygen depletion compound create an insulating interface. The extent of these effects can be modulated by applying an electric field to the device, which moves oxygen vacancies in and out of the interface depending on the polarity of the field. The movement of oxygen vacancies modifies the AlO_*x*_ layer and the oxygen deficiency level of the interfacial GCMO, which leads to nonvolatile hysteresis in the *I*(*V*) properties.^22^ Both the AlO_*x*_ layer and the formation of oxygen-deficient perovskite region have been observed in other manganite oxides.^24,29−32^

The switching in the devices is bipolar, where the high and low resistive states can be achieved by the application of opposite voltage polarities, similar to what has been reported for other manganite compounds.^1,17,22,24,33−36^ The voltage amplitudes for RS are asymmetrical, and the transition to the negative polarity-induced HRS happens at a lower amplitude than the positive polarity-induced LRS transition. The pristine state of the devices is close to the LRS. The switching between HRS and LRS happens gradually, which makes it possible to program intermediate states. The switching in the devices does not require a high-field forming step or current limitation, contrary to many other RS materials.

### Effect of Calcium Doping

The calcium substitution *x* greatly affects both the bulk and RS properties of the GCMO (Figure 2). The Au interface remains Ohmic over the whole substitution range and does not contribute to the switching. The resistivity of the Au interface is lower at higher *x*, with the minimum at *x* = 0.85. The resistance values of the Au interface coincide with the existing four-point measurements on GCMO thin films.^26^

**Figure 3:**
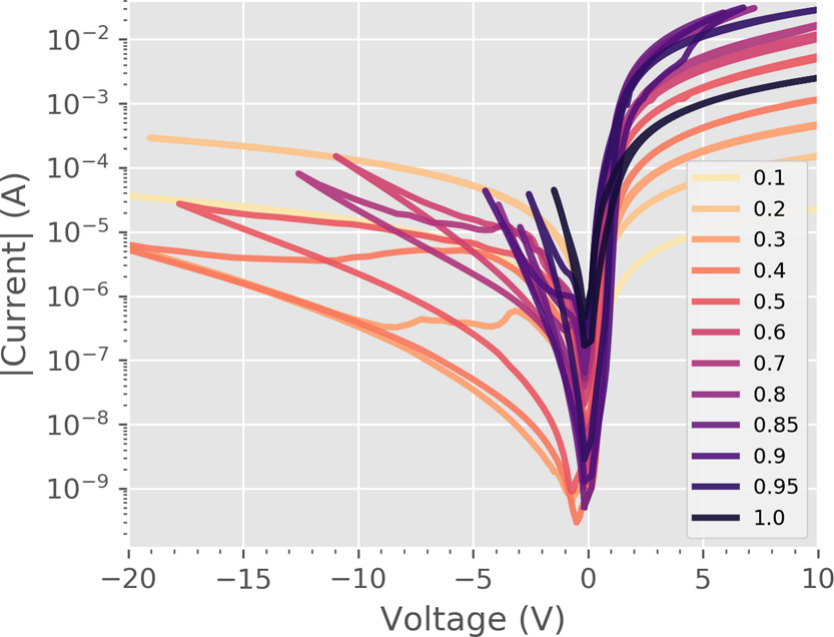
Averages of 50 repeated *I*(*V*) loops for each calcium substitution level. Required switching voltages decrease at higher *x*, and the switching becomes more asymmetric.

The averages of 50 stable *I*(*V*) loops of the Al interface as a function of calcium substitution level *x* are shown in Figure 3 and the corresponding switching ratios are shown in Figure 5. The switching ratio is defined as the ratio of stable minimum and maximum resistances measured at 450 mV, which is in the linear region and below the switching threshold for all concentrations. The switching is linked to the nonlinearity of the Al interface, since below *x* = 0.4, the samples show nearly Ohmic behavior and the switching is weak.

**Figure 4:**
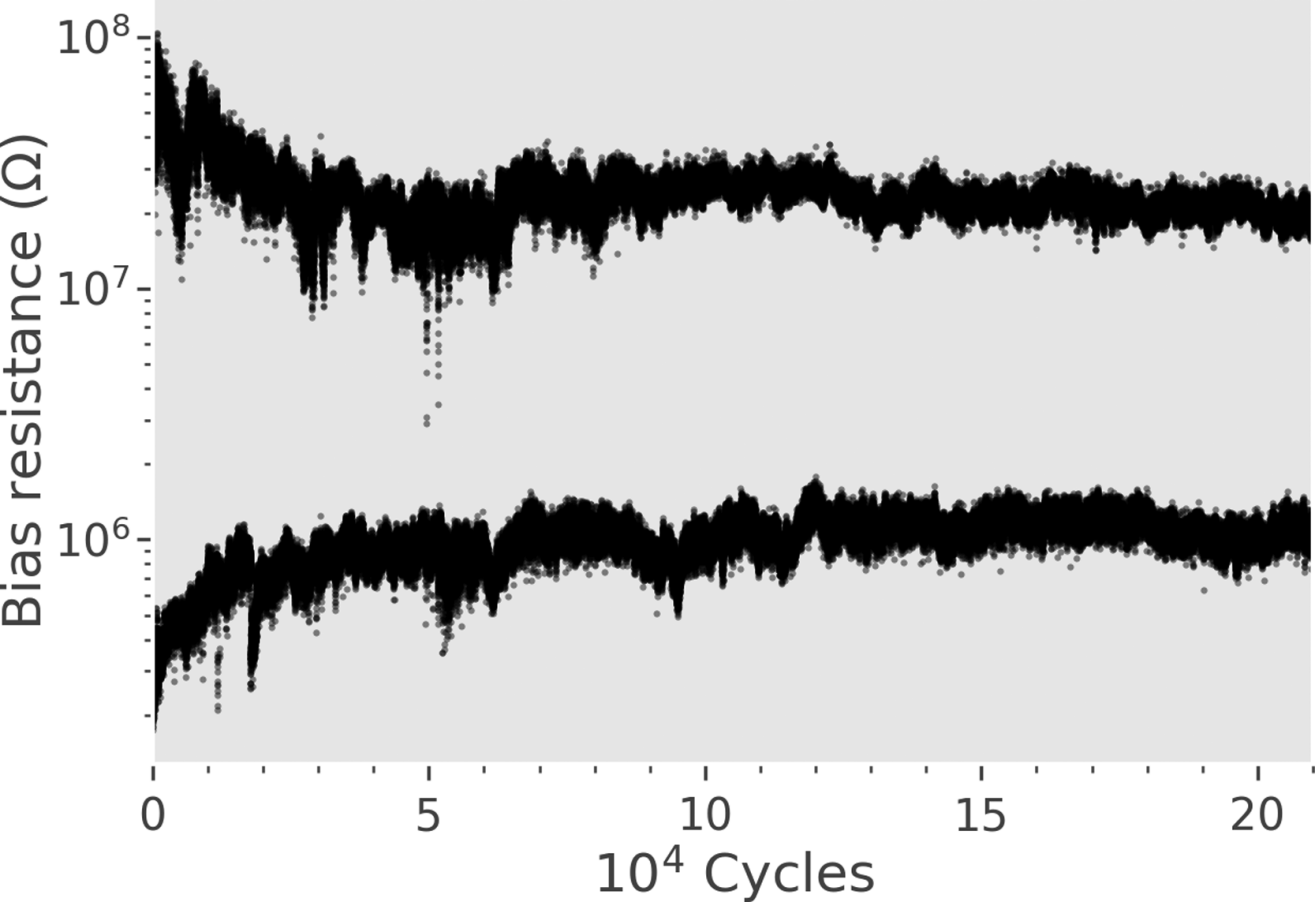
Device endurance of the calcium substitution *x* = 0.85 over 2 × 10^5^ repeated alternating HRS and LRS pulses. The reading was done between the pulses at the Ohmic region with 450 mV voltage amplitude.

**Figure 5:**
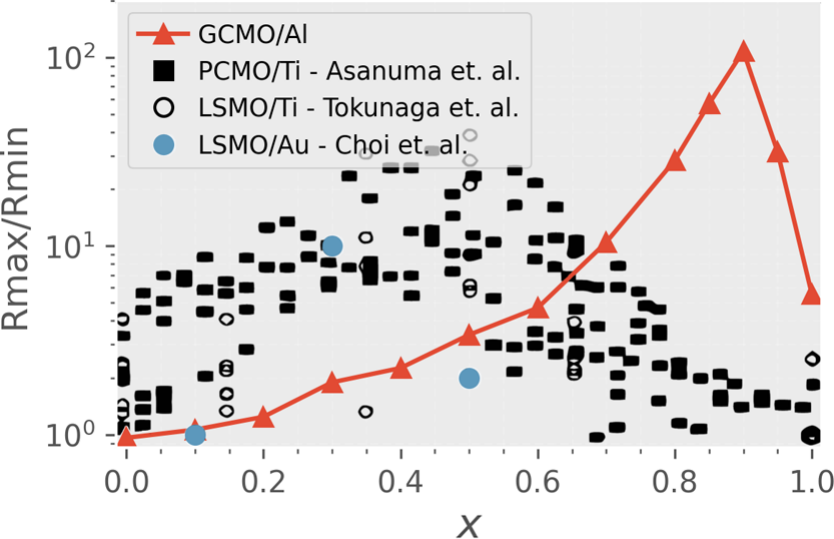
Switching ratio over *x* for Al/GCMO, Ti/PCMO,^24^ Ti/LSMO,^37^ and Au/LSMO.^38^ The ratio for GCMO was determined from after-pulse measurements of switching loops at 450 mV, where the samples were Ohmic. Results for Ti/LSMO reprinted with permission from^37^ Copyright 2006 AIP publishing. Results for Ti/PCMO reprinted with permission from^24^ Copyright 2009 American Physical Society. Results for Au/LSMO reprinted with permission from^38^ Copyright 2013 Elsevier.

The nonvolatile bipolar RS is present in the range 0.4 < *x* < 0.95, and the switching properties peak in the range 0.7 < *x* < 0.95. The concentrations with a low *x* have high bulk resistivity and require large voltage amplitudes to induce switching. The required switching voltages decrease, and the asymmetry in switching voltages increases at substitution levels above *x* = 0.7.

The optimal calcium concentration for RS was found to be *x* = 0.85, which is the point where the maximum HRS resistance, the lowest switching voltages, and the lowest bulk resistivity are reached (Figures 2 and 3). The optimal concentration of *x* = 0.85 was also confirmed to withstand over 2 × 10^5^ repeated cycles between HRS and LRS (Figure 4). The HRS remains above 10^7^ Ω in all switching samples, which is beneficial in reducing the sneak-path currents in crossbar arrays, although full mitigation requires additional measures.^39^

The range of calcium substitution where nonvolatile switching is present coincides with changes in the magnetic properties.^26^ The phase diagram has a gradual transition from insulating to metallic state (where the derivative of resistivity with respect to temperature is positive), which begins at *x* = 0.5 and ends at a region between *x* = 0.95 and *x* = 1.0, after which the bulk becomes insulating at *x* = 1.0. The conditions for magnetic charge ordering also set in at *x* = 0.4 and disappear approximately at *x* = 0.7, with effect being the strongest at half-doping.

### Other Manganites

In order to highlight the differences between GCMO and other manganite materials, the switching ratios obtained from Al/GCMO over the calcium concentration range *x* were compared with other studies on manganites (Figure 5). The switching ratio was used in the comparison, since absolute values, such as resistances and threshold voltages, depend on the size and geometry of the device.

The other materials used in the comparison were Ti/LSMO,^37^ Au/LSMO,^38^ and Ti/PCMO.^24^ The comparison was made against studies which included a series of devices made with different calcium concentrations, and this allowed the determination of the optimal *x* for each material/electrode combination. The authors of the Ti/LSMO study point out that the Al switching in LSMO is similar to the Ti switching with a slightly lesser switching ratio.^37^

The behavior of the GCMO is different from the LSMO and PCMO samples. What can be seen is that the optimal ratio is in a different part of the phase diagram. While LSMO and PCMO have the maximum at half-doping, the optimal region for the GCMO is at *x* = 0.9, a composition where the other materials do not show switching at all. This is remarkable since the compared materials share similar structural and magnetic properties, and the physical mechanism behind the RS process has been established as universal for the family of Mn-based devices.^1,22,40^

RS in PCMO-based devices has been extensively studied, and most works tend to agree that a moderate hole-carrier concentration of manganites is a prerequisite for the RS and that heavy hole doping suppresses the effect.^24^ Our results on GCMO indicate that we cannot generalize this conclusion to the whole manganite family-based devices. This unexpected result demonstrates that the understanding of the physics beneath this phenomenon is still incomplete, and RS materials are being neglected due to their similarity to those already reported.

### Conduction Model

The effects of *x* in the RS and conduction properties were studied in more detail by analyzing the conduction mechanism from the measured *I*(*V*) curves. The quantity γ = *d*(ln(|*I*|))/*d*(ln(|*V*|)) was utilized to analyze changes in the conduction exponent. Different nonlinear (NL) elements and their combinations^41^ produce distinct γ(*V*^1/2^) relations, which can be used to differentiate between different conduction models. An Ohmic interface (*I* ∝ *V*) will result in a constant γ = 1, while a space-charge limited conduction (*I* ∝ *V*^2^) dominated interface will have a constant of γ = 2. Conduction mechanisms that have an exponential dependence between current and voltage, such as Poole–Frenkel (PF) and Schottky, will present a straight line which will only differ in the *y*-intercept value (0 for Schottky and 1 for PF).^42^

**Figure 6:**
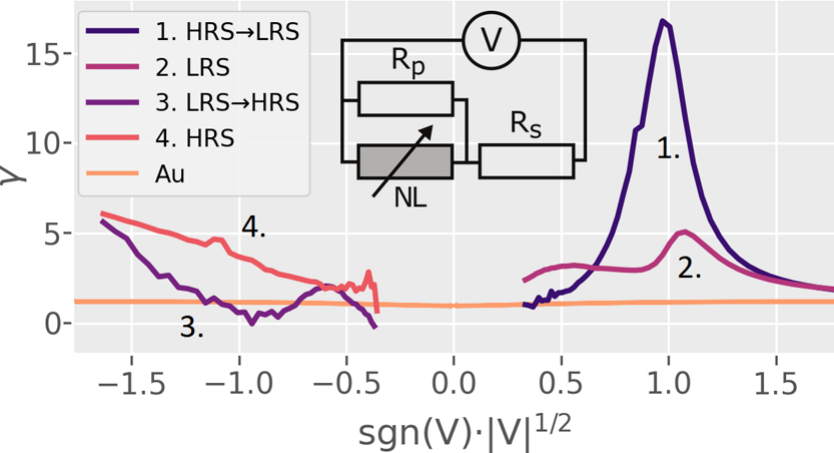
Voltage sweep γ(*V*^1/2^) curves of Ohmic Au interfaces and Al/GCMO/Au for *x* = 0.85. RS and nonlinear conduction happen only when an Al interface is present. The inset shows the equivalent circuit used in model fits, which consists of a NL element with series and parallel linear resistances *R*_s_ and *R*_p_.

However, since the electric transport in our devices is not dominated by a single conduction mechanism, γ(*V*^1/2^) will take on varying shapes (Figure 6) due to different dominant conduction mechanisms over the voltage range. The positive polarity has a peak-shaped γ-dependence in both HRS and LRS resulting from an interplay of PF and Ohmic conduction mechanisms. The γ-dependence of the negative polarity has two different modes of conduction depending on the resistive state. The change from LRS to HRS in the negative polarity γ initially mirrors the positive polarity, until the transition to HRS begins. In the negative HRS, the γ-relation changes into a Schottky-like conduction with a constant slope and a y-intercept of approximately 0. The simultaneous presence of both Schottky and PF conduction models has previous support.^43^ The negative polarity was not utilized in the final model analysis due to the highly rectifying interface and the stochastic transition from PF to Schottky-like conduction, which is difficult to reproduce using physical models.^44^

In order to properly explain the peak-shaped γ(*V*^1/2^) behavior in the positive polarity of GCMO, a more complex circuit needs to be introduced. The positive polarity curves of Al/GCMO can be reproduced by creating an equivalent circuit consisting of a NL conduction element with Ohmic series and parallel resistances *R*_s_ and *R*_p_ (Figure 6). The NL element representing the contribution of the Al/GCMO interface can be thought of as a bulk-limited PF conduction in series with a Schottky diode.^43^ The circuit can be used in the positive polarity, where the PF-based conduction dominates. A similar peak shape has also been seen in other perovskite compounds, such as PCMO^14^ and LSCO.^45^

The model can be expressed as an implicit equation for *I* and *V* at constant temperature
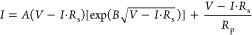
1with  and , where *T* is the temperature, *Ã*_PF_ is a normalization factor which also depends on temperature, *q* is the electron charge, ϕ is the trap energy level, *k*_B_ is the Boltzmann constant, ϵ_0_ is the vacuum permittivity, ϵ′ is the real part of the dielectric constant, *d* is the distance where the voltage drop is produced, *R*_s_ is the series resistance, and *R*_p_ is the parallel resistance.^42^ The series resistance *R*_s_ represents the Ohmic RS-state dependent contribution from the oxide layer and the bulk, that is, *R*_s_ is high in HRS and low in LRS. The parallel resistance *R*_p_ represents the Ohmic contribution from regions of the interface which do not contribute to the switching; an *R*_p_ higher than the bulk resistivity is a requirement for RS.

### Model Fit

The model makes it possible to separate Ohmic components from the positive *I*(*V*) curve and examine the underlying PF conduction, which combined with temperature measurements gives access to the PF trap energy level ϕ.

The fitting for free parameters *A*, *B*, *R*_s_, and *R*_p_ of eq 1 was done by numerically solving the implicit equation and iteratively minimizing the error with respect to the experimental data by using a combination of basin-hopping and Broyden–Fletcher–Goldfarb–Shanno algorithm. The data used for the fits was the nonrectifying positive polarity in the HRS. The HRS was used because there is a continuum of possible states between HRS and LRS, out of which HRS represents the maximal difference from the nonswitching Ohmic interface (Figure 6).

**Figure 7:**
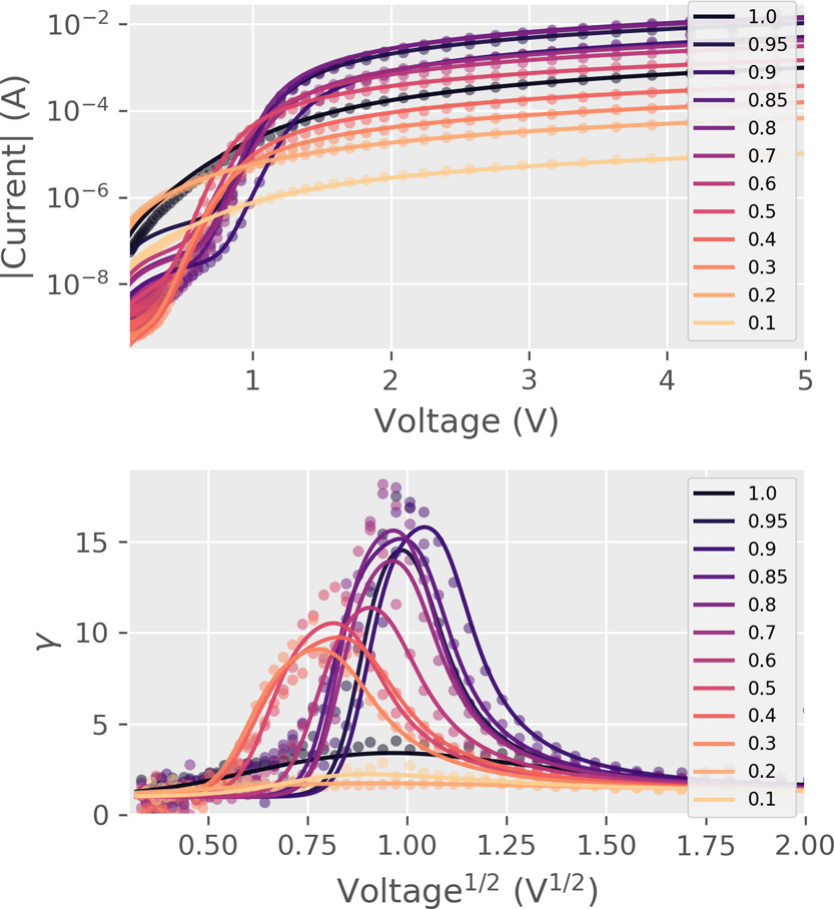
Fitted and experimental values for γ(*V*^1/2^) and *I*(*V*) over the calcium range at room temperature. The transition becomes more abrupt at higher substitutions.

The room-temperature fits were made for samples in the range 0.1 ≤ *x* ≤ 1.0. The model coincided with the experimental data at room temperature, reproducing the *I*(*V*) relations for voltages above 400 mV (Figure 7). The series resistances *R*_s_ coincide with the resistance of GCMO with Ohmic electrodes (Table 1) (Figure 2). The parallel resistances *R*_p_ were of the same order of magnitude as the HRS states in switching Al/GCMO interfaces (Figure 2).

**Table 1:** Table with Parameters *R*_s_, *R*_p_, and ϕ from the Model Fit and Experimentally Measured Resistances *R*_Au_ from Symmetric Au Interfaces at Room Temperature

*x*	*R*_s_ (Ω)	*R*_p_ (Ω)	ϕ (eV)	*R*_Au_ (Ω)
0.0				3.7 × 10^6^
0.1	3.6 × 10^5^	3.7 × 10^6^		1.9 × 10^5^
0.2	4.9 × 10^4^	8.3 × 10^5^		3.7 × 10^4^
0.3	2.4 × 10^4^	2.4 × 10^8^		8.9 × 10^3^
0.4	1.0 × 10^4^	2.8 × 10^8^	1.00	5.0 × 10^3^
0.5	2.6 × 10^3^	3.5 × 10^7^	0.99	1.3 × 10^3^
0.6	1.1 × 10^3^	6.8 × 10^6^	0.98	1.0 × 10^3^
0.7	8.4 × 10^2^	1.1 × 10^7^	0.85	4.2 × 10^2^
0.8	2.6 × 10^2^	9.9 × 10^6^	0.30	1.8 × 10^2^
0.85	2.4 × 10^2^	2.1 × 10^7^	0.44	1.4 × 10^2^
0.9	6.7 × 10^2^	2.1 × 10^7^	0.67	1.5 × 10^2^
0.95	3.3 × 10^2^	2.0 × 10^6^	0.70	1.7 × 10^2^
1.0	2.9 × 10^3^	1.1 × 10^6^		3.1 × 10^3^

### Temperature Dependence

**Figure 8:**
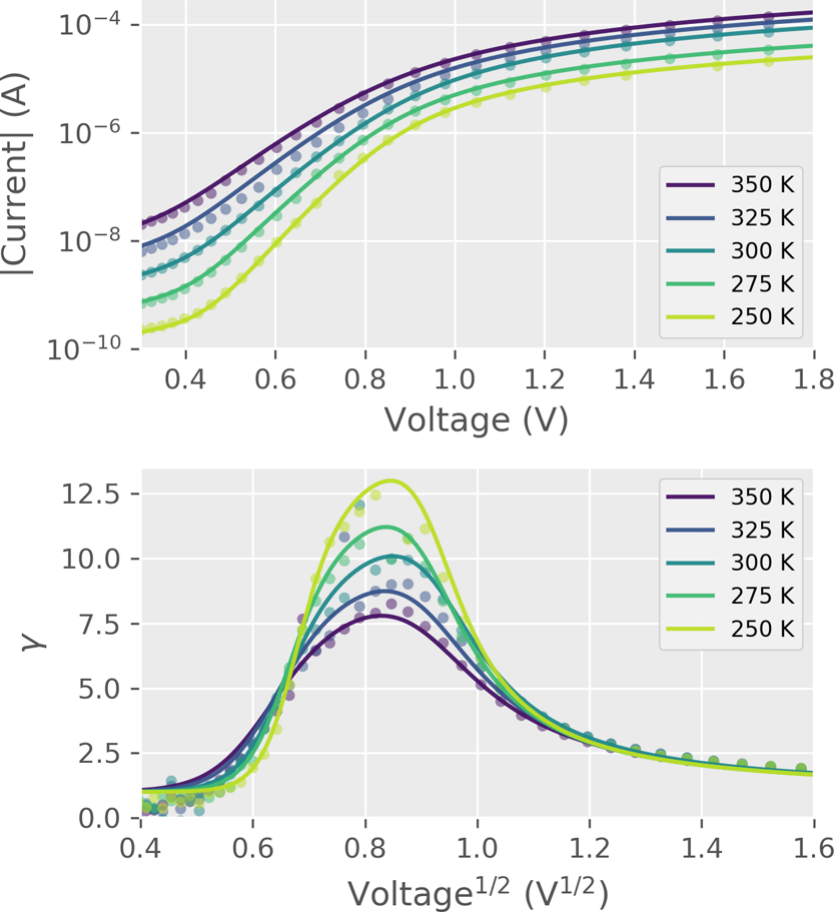
Fitted and experimental values for γ(*V*^1/2^) and *I*(*V*) for *x* = 0.4 over the temperature range.

**Figure 9:**
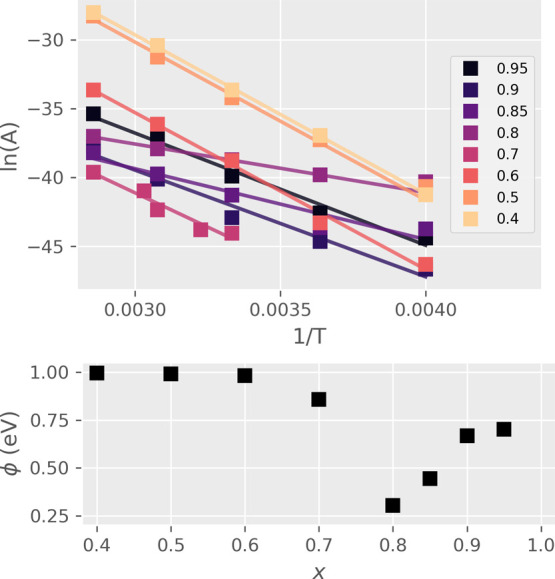
Arrhenius plots and PF trap energy levels ϕ from the model fit. The substitution values *x* where trap energy level ϕ deviates from 1 eV also correspond to good RS properties.

We determined the PF trap energy level ϕ by measuring the *I*(*V*) curves as a function of temperature. The measurements were performed in a physical property measurement system. The samples were set to the HRS at 300 K, after which they were measured over the temperature range with the Keithley 2614b sourcemeter. The *I*–*V* measurements were run using a maximum voltage amplitude, which did not induce switching at any temperature. The measurements consisted of initially going to the maximum temperature of 350 K, after which the temperature was lowered to 250 K in steps of 25 K. Experimental data from each temperature measurement was fitted to the conduction model (Figure 8). Fitting for ϕ was performed for concentrations which had RS and linear Arrhenius relation ln(*A*) – 1/*T*; this corresponded to the *x* range from 0.4 to 0.95 (Table 1) (Figure 9).

The trap energy level for *x* in the range from 0.4 to 0.6 is close to 1 eV. From 0.6 to 0.8, the value of ϕ decreases to 0.3 eV and begins to increase again at higher *x*. The lowest point of ϕ at *x* = 0.8 coincides with the region of highest switching ratio (Figure 5), which suggests that the magnitude of RS is related to changes in the trap energy level. The minimum also has the lowest threshold voltage needed for inducing HRS to LRS transition and corresponds to the lowest point in the bulk resistance of GCMO (Table 1) (Figure 2). The RS in Al/GCMO seems to favor low bulk resistance and shallow traps at the active interface. These findings could be useful as heuristics for finding optimal RS parameters in other similar materials.

## Conclusions

In summary, we have demonstrated that the RS effect is present in the Al/Gd_1–*x*_Ca_*x*_MnO_3_-interface at certain calcium substitution values. The devices showed bipolar switching when combined with an asymmetric combination of Ohmic interface and a rectifying Al interface. The optimum concentration for RS with operating voltages and switching ratio taken into account was determined to be near *x* = 0.9. This conclusion completely opposes the behavior in similar manganites, where the optimal value is close to half-doping and no switching is observable at high substitution values.

The positive polarity of Al/GCMO was found to obey PF conduction with linear series and parallel resistances and the negative polarity HRS conduction was Schottky-like. The temperature measurements showed changes in the PF trap energy ϕ in the range from 0.3 to 1.00 eV. The lower ϕ values were associated with good RS properties, high switching ratio, and low bulk resistivity.

These RS studies show that the GCMO-based devices are on par with the other manganite-based RS materials and open the question on whether the other unexplored members of the manganite family could be used for RS as well. The next step in the GCMO-based memristor devices is to use the knowledge gained from our research to optimize the devices up to their fullest potential and to study their properties from the neuromorphics standpoint. This will lead to the development of new technologies in the form of more robust and energy-efficient memory devices and neuromorphic circuits.

## References

[ref1] Sawa A. Resistive Switching in Transition Metal Oxides. Mater. Today 2008, 11, 28–36. 10.1016/s1369-7021(08)70119-6.

[ref2] Yoon J. H.; Han J. H.; Jung J. S.; Jeon W.; Kim G. H.; Song S. J.; Seok J. Y.; Yoon K. J.; Lee M. H.; Hwang C. S. Highly Improved Uniformity in the Resistive Switching Parameters of TiO_2_ Thin Films by Inserting Ru Nanodots. Adv. Mater. 2013, 25, 1957 10.1002/adma.201204572. 23386379

[ref3] Kwon D.-H.; Kim K. M.; Jang J. H.; Jeon J. M.; Lee M. H.; Kim G. H.; Li X.-S.; Park G.-S.; Lee B.; Han S.; Kim M.; Hwang C. S. Atomic Structure of Conducting Nanofilaments in TiO_2_ Resistive Switching Memory. Nat. Nanotechnol. 2010, 5, 148 10.1038/nnano.2009.456. 20081847

[ref4] Shi Y.; Liang X.; Yuan B.; Chen V.; Li H.; Hui F.; Yu Z.; Yuan F.; Pop E.; Wong H.-S. P.; Lanza M. Electronic Synapses Made of Layered Two-Dimensional Materials. Nat. Electron. 2018, 1, 458 10.1038/s41928-018-0118-9.

[ref5] Ishibe T.; Kurokawa T.; Naruse N.; Nakamura Y. Resistive Switching at the High Quality metal/insulator Interface in Fe_3_O_4_/SiO_2_/*α*-FeSi_2_/Si Stacking Structure. Appl. Phys. Lett. 2018, 113, 141601 10.1063/1.5048827.

[ref6] Ishibe T.; Maeda Y.; Terada T.; Naruse N.; Mera Y.; Kobayashi E.; Nakamura Y. Resistive Switching Memory Performance in Oxide Hetero-Nanocrystals With Well-Controlled Interfaces. Sci. Technol. Adv. Mater. 2020, 21, 195 10.1080/14686996.2020.1736948. 32284769 PMC7144302

[ref7] Zahoor F.; Azni Zulkifli T. Z.; Khanday F. A. Resistive Random Access Memory (RRAM): an Overview of Materials, Switching Mechanism, Performance, Multilevel Cell (MLC) Storage, Modeling, and Applications. Nanoscale Res. Lett. 2020, 15, 90 10.1186/s11671-020-03299-9. 32323059 PMC7176808

[ref8] Lee D.; Hwang H. Neuro-inspired Computing Using Resistive Synaptic Devices. Pr_0.7_Ca_0.3_MnO_3_ (PCMO)-Based Synaptic Devices; Springer, 2017; pp 53–71.

[ref9] Lashkare S.; Chouhan S.; Chavan T.; Bhat A.; Kumbhare P.; Ganguly U. PCMO RRAM for Integrate-And-Fire Neuron in Spiking Neural Networks. IEEE Electron Device Lett. 2018, 39, 484–487. 10.1109/led.2018.2805822.

[ref10] Li C.; et al. Analogue Signal and Image Processing With Large Memristor Crossbars. Nat. Electron. 2018, 1, 52 10.1038/s41928-017-0002-z.

[ref11] Yao P.; Wu H.; Gao B.; Tang J.; Zhang Q.; Zhang W.; Yang J. J.; Qian H. Fully Hardware-Implemented Memristor Convolutional Neural Network. Nature 2020, 577, 641–646. 10.1038/s41586-020-1942-4. 31996818

[ref12] Gao L.; Chen P.-Y.; Yu S. Demonstration of Convolution Kernel Operation on Resistive Cross-Point Array. IEEE Electron Device Lett. 2016, 37, 870–873. 10.1109/led.2016.2573140.

[ref13] Park S. RRAM-Based Synapse for Neuromorphic System with Pattern Recognition Function, 2012; International Electron Devices Meeting, 2012; pp 10–12.

[ref14] Lähteenlahti V.; Schulman A.; Huhtinen H.; Paturi P. Transport Properties of Resistive Switching in Ag/Pr_0.6_Ca_0.4_MnO_3_/Al Thin Film Structures. J. Alloys Compd. 2019, 786, 84–90. 10.1016/j.jallcom.2019.01.279.

[ref15] Jang J.-W.; Park S.; Burr G. W.; Hwang H.; Jeong Y.-H. Optimization of Conductance Change in Pr_1–*x*_Ca_*x*_MnO_3_-based Synaptic Devices for Neuromorphic Systems. IEEE Electron Device Lett. 2015, 36, 457–459. 10.1109/led.2015.2418342.

[ref16] Zhao M.; Gao B.; Tang J.; Qian H.; Wu H. Reliability of Analog Resistive Switching Memory for Neuromorphic Computing. Appl. Phys. Rev. 2020, 7, 011301 10.1063/1.5124915.

[ref17] Bagdzevicius S.; Maas K.; Boudard M.; Burriel M. Interface-Type Resistive Switching in Perovskite Materials. J. Electroceram. 2017, 39, 157–184. 10.1007/s10832-017-0087-9.

[ref18] Kanegami N.; Nishi Y.; Kimoto T. Unique Resistive Switching Phenomena Exhibiting both Filament-Type and Interface-Type Switching in Ti/Pr_0.7_Ca_0.3_MnO3- *δ*/Pt ReRAM Cells. Appl. Phys. Lett. 2020, 116, 013501 10.1063/1.5131090.

[ref19] Park S.; Sheri A.; Kim J.; Noh J.; Jang J.; Jeon M.; Lee B.; Lee B.; Lee B.; Hwang H.-J. Neuromorphic Speech Systems Using Advanced Reram-Based Synapse, 2013; IEEE IEDM, 2013; pp 25–26.

[ref20] Miranda E.; Román Acevedo W.; Rubi D.; Lüders U.; Granell P.; Suñé J.; Levy P. Modeling of the Multilevel Conduction Characteristics and Fatigue Profile of Ag/La_1/3_Ca_2/3_MnO_3_/Pt Structures Using a Compact Memristive Approach. J. Appl. Phys. 2017, 121, 205302 10.1063/1.4984051.

[ref21] Ortega-Hernandez R.; Coll M.; Gonzalez-Rosillo J.; Palau A.; Obradors X.; Miranda E.; Puig T.; Suñe J. Resistive Switching in CeO_2_/La_0.8_Sr_0.2_MnO_3_ Bilayer for Non-Volatile Memory Applications. Microelectron. Eng. 2015, 147, 37–40. 10.1016/j.mee.2015.04.042.

[ref22] Rozenberg M.; Sanchez M. J.; Weht R.; Acha C.; Gomez-Marlasca F.; Levy P. Mechanism for Bipolar Resistive Switching in Transition-Metal Oxides. Phys. Rev. B: Condens. Matter Mater. Phys. 2010, 81, 115101 10.1103/physrevb.81.115101.

[ref23] Lee D.; Park J.; Moon K.; Jang J.; Park S.; Chu M.; Kim J.; Noh J.; Jeon M.; Lee B. H.; Lee B.; Lee B.-G.; Hyunsang H. Oxide Based Nanoscale Analog Synapse Device for Neural Signal Recognition System, 2015; IEEE International Electron Device Meeting, 2015; pp 4–7.

[ref24] Asanuma S.; Akoh H.; Yamada H.; Sawa A. Relationship Between Resistive Switching Characteristics and Band Diagrams of Ti/Pr_1–*x*_Ca_*x*_MnO_3_ Junctions. Phys. Rev. B: Condens. Matter Mater. Phys. 2009, 80, 235113 10.1103/physrevb.80.235113.

[ref25] Beiranvand A.; Tikkanen J.; Huhtinen H.; Paturi P. Electronic and Magnetic Phase Diagram of Polycrystalline Gd_1–*x*_Ca_*x*_MnO_3_ Manganites. J. Alloys Compd. 2017, 720, 126–130. 10.1016/j.jallcom.2017.05.231.

[ref26] Schulman A.; Beiranvand A.; Lähteenlahti V.; Huhtinen H.; Paturi P. Appearance of Glassy Ferromagnetic Behavior in Gd_1–*x*_Ca_*x*_MnO_3_ (0 < x < 1) Thin Films: A Revised Phase Diagram. J. Magn. Magn. Mater. 2020, 498, 166149 10.1016/j.jmmm.2019.166149.

[ref27] Paturi P.; Tikkanen J.; Huhtinen H. Room Temperature Charge-Ordered Phase in Gd_0.6_Ca_0.4_MnO_3_ and Sm_0.6_Ca_0.4_MnO_3_ Thin Films. J. Magn. Magn. Mater. 2017, 432, 164–168. 10.1016/j.jmmm.2017.01.080.

[ref28] Beiranvand A.; Tikkanen J.; Huhtinen H.; Paturi P. Metamagnetic Transition and Spin Memory Effect in Epitaxial Gd_1–*x*_Ca_*x*_MnO_3_ (0 < x < 1) Thin Films. J. Magn. Magn. Mater. 2019, 469, 253–258. 10.1016/j.jmmm.2018.08.002.

[ref29] Liao Z. L.; Wang Z. Z.; Meng Y.; Liu Z. Y.; Gao P.; Gang J. L.; Zhao H. W.; Liang X. J.; Bai X. D.; Chen D. M. Categorization of Resistive Switching of metal-Pr_0.7_Ca_0.3_MnO_3_-metal Devices. Appl. Phys. Lett. 2009, 94, 253503 10.1063/1.3159471.

[ref30] Yang R.; Li X. M.; Yu W. D.; Liu X. J.; Cao X.; Wang Q.; Chen L. D. Multiform Resistance Switching Effects in the Al/La_0.7_Ca_0.3_MnO_3_/Pt Structure. Electrochem. Solid State Lett. 2009, 12, H281–H283. 10.1149/1.3129136.

[ref31] Seong D.-J.; Hassan M.; Choi H.; Lee J.; Yoon J.; Park J.-B.; Lee W.; Oh M.-S.; Hwang H. Resistive-Switching Characteristics of Al/Pr_0.7_Ca_0.3_MnO_3_ for Nonvolatile Memory Applications. IEEE Electron Device Lett. 2009, 30, 919–921. 10.1109/LED.2009.2025896.

[ref32] Nakamura T.; Homma K.; Tachibana K. Thin Film Deposition of Metal Oxides in Resistance Switching Devices: Electrode Material Dependence of Resistance Switching in Manganite Films. Nanoscale Res. Lett. 2013, 8, 76 10.1186/1556-276x-8-76. 23414549 PMC3577670

[ref33] Baek K.; Park S.; Park J.; Kim Y.-M.; Hwang H.; Oh S. H. In Situ Tem Observation on the Interface-Type Resistive Switching by Electrochemical Redox Reactions at a TiN/PCMO Interface. Nanoscale 2017, 9, 582–593. 10.1039/c6nr06293h. 27886327

[ref34] Kramer T.; Scherff M.; Mierwaldt D.; Hoffmann J.; Jooss C. Role of Oxygen Vacancies for Resistive Switching in Noble Metal Sandwiched Pr_0.67_Ca_0.33_MnO_3_-*δ*. Appl. Phys. Lett. 2017, 110, 243502 10.1063/1.4985645.

[ref35] Seong T.-G.; Lee B.-S.; Choi K. B.; Kweon S.-H.; Kim B. Y.; Jung K.; Moon J. W.; Lee K. J.; Hong K.; Nahm S. Unipolar Resistive Switching Properties of Amorphous Pr_0.7_Ca_0.3_MnO_3_ Films Grown on a Pt/Ti/SiO_2_/Si Substrate. Curr. Appl. Phys. 2014, 14, 538–542. 10.1016/j.cap.2014.01.012.

[ref36] Toyoda S.; Namiki T.; Sakai E.; Nakata K.; Oshima M.; Kumigashira H. Chemical-State-Resolved Depth Profiles of Al/Pr_0.7_Ca_0.3_MnO_3_ Stacked Structures for Application in Resistive Switching Devices. J. Appl. Phys. 2013, 114, 243711 10.1063/1.4858381.

[ref37] Tokunaga Y.; Kaneko Y.; He J. P.; Arima T.; Sawa A.; Fujii T.; Kawasaki M.; Tokura Y. Colossal Electroresistance Effect at Metal electrode/La_1–*x*_Sr_1+*x*_MnO_4_ Interfaces. Appl. Phys. Lett. 2006, 88, 223507 10.1063/1.2208922.

[ref38] Choi S. G.; Lee H.-S.; Choi H.; Chung S.-W.; Park H.-H. The Effect of Sr Concentration on Resistive Switching Properties of La_1–*x*_Sr_*x*_MnO_3_ Films. Thin Solid Films 2013, 529, 352–355. 10.1016/j.tsf.2012.07.069.

[ref39] Zidan M. A.; Fahmy H. A. H.; Hussain M. M.; Salama K. N. Memristor-Based Memory: The Sneak Paths Problem and Solutions. Microelectron. J. 2013, 44, 176–183. 10.1016/j.mejo.2012.10.001.

[ref40] Waser R.; Dittmann R.; Staikov G.; Szot K. Redox-Based Resistive Switching Memories - Nanoionic Mechanisms, Prospects, and Challenges. Adv. Mater. 2009, 21, 2632–2663. 10.1002/adma.200900375. 36751064

[ref41] Chiu F.-C. A Review on Conduction Mechanisms in Dielectric Films. Adv. Mater. Sci. Eng. 2014, 2014, 578168 10.1155/2014/578168.

[ref42] Acha C. Graphical Analysis of Current-Voltage Characteristics in Memristive Interfaces. J. Appl. Phys. 2017, 121, 134502 10.1063/1.4979723.

[ref43] Lau W. S. An Extended Unified Schottky-Poole-Frenkel Theory to Explain the Current-Voltage Characteristics of Thin Film Metal-Insulator-Metal Capacitors With Examples for Various High-K Dielectric Materials. ECS J. Solid State Sci. Technol. 2012, 1, N139 10.1149/2.006301jss.

[ref44] Aguirre F. L.; Pazos S. M.; Palumbo F.; Suñé J.; Miranda E. Application of the Quasi-Static Memdiode Model in Cross-Point Arrays for Large Dataset Pattern Recognition. IEEE Access 2020, 8, 202174–202193. 10.1109/access.2020.3035638.

[ref45] Acha C.; Schulman A.; Boudard M.; Daoudi K.; Tsuchiya T. Transport Mechanism Through Metal-Cobaltite Interfaces. Appl. Phys. Lett. 2016, 109, 011603 10.1063/1.4955204.

